# Size-Dependent Effects of Gold Nanoparticles Uptake on Maturation and Antitumor Functions of Human Dendritic Cells In Vitro

**DOI:** 10.1371/journal.pone.0096584

**Published:** 2014-05-06

**Authors:** Sergej Tomić, Jelena Đokić, Saša Vasilijić, Nina Ogrinc, Rebeka Rudolf, Primož Pelicon, Dragana Vučević, Petar Milosavljević, Srđa Janković, Ivan Anžel, Jelena Rajković, Marjan Slak Rupnik, Bernd Friedrich, Miodrag Čolić

**Affiliations:** 1 Medical Faculty of the Military Medical Academy, University of Defense, Belgrade, Serbia; 2 Institute for Medical Research of the Military Medical Academy, University of Defense, Belgrade, Serbia; 3 Microanalytical Center, Jožef Stefan Institute, Ljubljana, Slovenia; 4 LOTRIČ Metrology, Selca, Slovenia; 5 Faculty of Mechanical Engineering, University of Maribor, Maribor, Slovenia; 6 Zlatarna Celje d.d., Celje, Slovenia; 7 University Children's Hospital, Belgrade, Serbia; 8 Medical Faculty, University of Niš, Niš, Serbia; 9 Medical Faculty, University of Maribor, Maribor, Slovenia; 10 Department of Process Metallurgy and Metal Recycling, Rheinisch-Westfälische Technische Hochschule, Aachen University, Aachen, Germany; National Institute of Health (NIH), United States of America

## Abstract

Gold nanoparticles (GNPs) are claimed as outstanding biomedical tools for cancer diagnostics and photo-thermal therapy, but without enough evidence on their potentially adverse immunological effects. Using a model of human dendritic cells (DCs), we showed that 10 nm- and 50 nm-sized GNPs (GNP_10_ and GNP_50_, respectively) were internalized predominantly via dynamin-dependent mechanisms, and they both impaired LPS-induced maturation and allostimulatory capacity of DCs, although the effect of GNP_10_ was more prominent. However, GNP_10_ inhibited LPS-induced production of IL-12p70 by DCs, and potentiated their Th2 polarization capacity, while GNP_50_ promoted Th17 polarization. Such effects of GNP_10_ correlated with a stronger inhibition of LPS-induced changes in Ca^2+^ oscillations, their higher number per DC, and more frequent extra-endosomal localization, as judged by live-cell imaging, proton, and electron microscopy, respectively. Even when released from heat-killed necrotic HEp-2 cells, GNP_10_ inhibited the necrotic tumor cell-induced maturation and functions of DCs, potentiated their Th2/Th17 polarization capacity, and thus, impaired the DCs' capacity to induce T cell-mediated anti-tumor cytotoxicity *in vitro*. Therefore, GNP_10_ could potentially induce more adverse DC-mediated immunological effects, compared to GNP_50_.

## Introduction

Spontaneous anti-tumor T cell-mediated immune response upon radio- or chemo-therapy is associated with a favorable prognosis [1]. Toll-like receptor (TLR)4^+^ dendritic cells (DCs) are described as key factors regulating the initiation of such a response, since the release of endogenous adjuvant from dying tumor cells stimulates the maturation of DCs via TLR4 [2], [3]. DCs are the most important professional antigen-presenting cells (APCs) regulating the development of adaptive anti-tumor response by presenting tumor antigens to T cells in an immunogenic or tolerogenic context [4]. Immature DCs take up and process antigens efficiently, but cannot prime T cells efficiently, leading to T cell anergy [5]. In contrast, activation of DCs' by lipopolysaccharide (LPS) [6] or high mobility group box (HMGB)-1 [3] via TLR4, leads to their phenotypic maturation and production of immune polarizing cytokines, such as IL-12p70, IL-23 [7], or IL-10 [5]. The signaling pathways leading to maturation of DCs include the translocation of Ca^2+^-sensitive transcription factors, such as NF-κB and NFAT, through nucleopores. The regulation of this translocation was found to be dependent on spontaneous Ca^2+^ oscillations, which are present in immature DCs, but not in DCs stimulated to undergo maturation [8].

Gold nanoparticles (GNPs) are recognized as very perspective diagnostic and therapeutic agents in cancer fighting strategies. They are easy to functionalize with drugs, genes and biomolecules [9]–[12], and are generally described as biocompatible, which supports their potential use in diagnostics [13]. However, some reports suggest that the endocytosed GNPs may cause cytotoxic effects, which could be beneficial for the treatment of cancer [14] and inflammatory/autoimmune diseases. The size- and shape-dependent optical properties of GNPs conferred to their surface Plasmon resonance [13], have already been exploited in imaging [15] and bio-sensing [16]. Furthermore, the ability of GNPs to convert near-infrared light efficiently into thermal energy upon absorption is being utilized for the development of targeted photo-thermal [17], or radio-sensitization cancer therapy [18].

However, it is still uncertain how the applied GNPs will alter the functions of immune cells, particularly DCs, upon application. *In vivo* investigations reported adjuvant properties of GNPs, and the involvement of Kupffer and Langerhans cells was implicated, but not clarified [19]. Once in circulation, GNPs were shown to be internalized by APCs via multiple routes [20], [21], all of which include dynamin-dependent mechanisms [22]. However, the distribution of GNPs upon internalization, which crucially determines the cellular response to them [23], [24], is still unclear and requires new methods of investigations, such as focused ion beam/scanning electron microscopy (FIB/SEM) [25]. Additionally, contradictory data exists on the immunological effects of GNPs once internalized by APCs. GNPs were shown to induce either pro-inflammatory [26] or anti-inflammatory effects [27], [28], depending on their size, conjugation and hydrophobicity [29]. Recently we showed, using a model of a mitogen stimulated rat's splenocytes, that bare GNPs, although non-cytotoxic [30], possess immunosuppressive properties [31]. Since these effects depend exclusively on the presence of APCs within the splenocytes' population [32], we hypothesized that GNPs could actually suppress the maturation of DCs, causing their impaired ability to trigger the antitumor response. In the present study, we showed how differently sized GNPs, applied at the non-toxic concentrations, interfere with the maturation and antitumor functions of DCs induced either by LPS or heat-killed necrotic cancer cells, and how these immunomodulatory effects correlate with GNPs uptake, their intracellular distribution, and their effects on Ca^2+^ signaling in DCs.

## Materials and Methods

### Cells

Peripheral blood mononuclear cells (PBMCs) of healthy donors (Table 1) were isolated in RPMI/0.02% NaEDTA on Lymphoprep gradient (PAA Laboratories) by density centrifugation (2200 rpm, 20 min, 20°C). Subsequently, PBMCs were used for the isolation of monocytes (n = 10) or CD3^+^ T cells (n = 9), which were negatively sorted by MACS using the Human Monocytes Isolation Kit II or Pan-T cell Isolation Kits (Miltenyi Biotec, Bergisch Gladbach, Germany) respectively. The purity of the cells was always higher than 90%, as judged by flow cytometry after the staining of cells with anti-CD14 and anti-CD3 antibodies, respectively. Immature DCs were generated by cultivating monocytes (0.5×10^6^/ml) in complete RPMI 1640 medium (10% FCS, 2 mM L-glutamine, 50 µM 2-mercapthoethanol (Sigma), penicillin/streptomycin/gentamicin, 1% each (ICN, Costa Mesa, CA, USA) with 100 ng/ml of human recombinant granulocyte-macrophage colony stimulating factor (GM-CSF) (Leucomax, Basel, Switzerland) and 20 ng/ml of the human recombinant interleukin (IL)-4 for 6 days, as described [33]. Immature DCs, identified by flow cytometry as CD1a^dim or bright^ CD14^−^ HLA-DR^+^ cells, were harvested and used in subsequent experiments.

**Table 1:** Demographic characteristics of healthy volunteers who provided PBMCs.

Total number of volunteers	19
Males number (%)	14 (73.6%)
Mean age (range)	33 (24–52)
Ethnicity (%)	European (100)

The blood was collected at the Institute of Blood Transfusion and Hemobiology of the Military Medical Academy, Belgrade, Serbia.

HEp-2, larynx epidermoid carcinoma cells were obtained from the American Type Culture Collection (Rockwell, MD, USA). The cells were plated at a density of 5,000 cells/cm^2^ and cultured in complete RPMI medium until they reached 70% confluence, after which passaging was performed by trypsinization.

### Ethics statements

Human PBMCs were isolated from healthy donors who signed Consent Forms, and the subjects' identities were kept confidential. All experiments were approved by the Ethical Board of the Military Medical Academy, Belgrade, Serbia (permission date: September 12^th^, 2012 in Belgrade), and the original documents are available upon request.

### Cell cultures

Immature DCs (0.5×10^6^/ml) were allowed to adhere for 2 h, and then spherical gold nanoparticles (Nanopartz Inc., CO, USA), 10 nm or 50 nm in size (GNP_10_ and GNP_50_, respectively) were added to the cultures (Table 2). GNPs were added in different concentrations (5–200 µg/ml of Au), followed by incubation at 37°C, 5% CO_2_ and 90% humidity for 4–48 h. Maturation of DCs was triggered by a TLR4 agonist, LPS from E. coli 0.111:B4 (Sigma, 100 ng/ml) for 48 h. In some experiments, LPS (100 ng/ml) was incubated with GNPs (10 or 50 µg/ml), or without them, in complete RPMI medium for 48 h, followed by centrifugation at 2000 g for 10 min. Control suspension was incubated likewise without LPS. Supernatant was collected by aspiration, and the pellet was re-suspended and washed two more times in complete RPMI medium. DCs were afterwards cultivated for 48 h in the supernatant, or in the washed pellet preparation which either contained the sonicated GNPs (10 or 50 µg/ml) or not. Alternatively, the maturation of DCs was induced by necrotic HEp-2 cells (ATCC, Rockwell, MD, USA) (1×10^6^/ml), that were previously treated with GNP_10_ or GNP_50_ (10 µg/ml each) for 24 h. After the culture, HEp-2 cells were collected and washed at 300 g for 8 min, and then heat-killed in complete RPMI medium at 63°C for 30 min, as described [34], which is similar to the temperatures achieved in GNP-based photo-thermal therapy [35]. After each treatment of DCs or HEp-2 cells, the cytospins were made (1×10^4^ cells/sample), and stained with May-Grunwald-Giemsa (MGG), or used for immunocytochemistry analysis. Supernatants collected in DCs' cultures were centrifuged at 2000 g for 10 min, and frozen at −40°C prior to the cytokines analysis. Supernatants from cell-free cultures, containing the same concentrations of GNPs, were prepared similarly and used as blank controls.

**Table 2:** Physicochemical properties of GNPs in water and medium.

Property	GNP_10_	GNP_50_
Size (nm)*	10	50
SRP peak (nm)*	532	535
Carboxylic acid-based capping agent (pending patent) (Da)*	<300	<300
Core size (TEM) (nm)	11.3±2.5 (n = 200)	51.9±3.7 (n = 200)
Hydrodynamic size (DLS) (nm)	Water: 10.2±0.8	Water: 50.9±0.7
	Medium: 17.6±1.8	Medium:73.8±0.8
PDI (DLS) (nm)	Water: 0.173±0.02	Water: 0.084±0.01
	Medium: 0.327±0.05	Medium: 0.278±0.02
z-potential (mV)	Water: −23.775±0.547	Water: −30.375±0.642
	Medium:−12.835±2.203	Medium: −12.250±1.147
Au concentrations in stock solutions (ICP-MS) (µg/ml)	2500	2500

*- Claimed by the vendor.

The concentration of Au within the GNP stock solutions was determined by Agilent Technologies 7500 ce ICP-MS, and it corresponded to the concentrations of Au reported by the vendor. The samples of GNPs (20 µg/ml) were prepared in DI 18 MEΩ water or complete medium, and sonicated for 20 s before the analysis by TEM or dynamic light scattering (DLS). The core size was analyzed on the preparations of GNPs added dropwise on the Cu-based grids and dried at room temperature, before the observation by JOEL JEM-2100 HR TEM (Tokyo, Japan) at 100 kV. The core size was similar in water and medium, so the average value from the measurements of GNPs in both solutions is shown as mean ± SD. Hydrodynamic size, polydispersion index (PDI), and z-potential in water and medium were analyzed by Zetasizer Nano ZS with non-invasive backscatter optics (NIBS). The samples were pre-warmed to 25°C for 120 s, and then analyzed for 15 min per run, for a total of 4 runs per sample, and the results are shown as mean ± SD (4).

### Mixed leukocytes reactions

The allostimulatory potential of DCs was assessed in co-culture with MACS purified allogeneic CD3^+^ T lymphocytes isolated from PBMCs. CD3^+^T cells (1×10^5^/well of 96-well plate) were co-cultivated with different numbers of DCs (1×10^4^, 0.5×10^4^ and 0.25×10^4^) for 5 days. The cytokines were detected in the supernatants of parallel DC/CD3^+^T cell co-cultures that were treated with PMA (20 ng/ml) and A23187 (500 ng/ml) (Sigma, Munich, Germany) for the last 8 h. After the co-cultures, supernatants were collected, centrifuged at 2000 g for 10 min, and frozen at −40°C prior to the analysis of cytokines, whereas the cells were counted and the levels of cytokines were normalized to 1×10^5^ cells/sample. Proliferation assays, performed in six replicates, were pulsed with 3H-thymidine for the last 18 h (1 µCi/well, Amersham Books, UK) and the radioactivity was measured by β-scintillation counting (LKB-1219 Rackbeta, Finland). The results are presented as counts per minute (CPM). Control cultures for proliferation and cytokine assays included CD3^+^T cells and corresponding DCs cultivated alone.

Additionally, CD3^+^T cells were primed with immature control DCs, or those matured with necrotic GNPs-treated or GNP-untreated HEp-2 cells for 3 days. The primed CD3^+^ T cells were then MACS-purified and expanded with IL-2 (2 ng/ml, R&D) for 2 days. T-cell cytotoxic activity was assessed by cultivating the primed CD3^+^T cells (2×10^4^, 4×10^4^, 8×10^4^) with live HEp-2 cells (1×10^4^) for 24 h. The cell cultures performed in six replicates were then treated with 3-[4,5 dimethyl-thiazol-2 lyl]- 2.5 diphenyl tetrazolium bromide (MTT) (Sigma, 100 µg/ml) for 4 h, and 0.1 N HCl/10% sodium dodecyl sulphate (SDS) overnight. Cell-free cultures were used as blank controls. The absorbance was read at 570/650 nm (ELISA reader, Behring II), and the values measured in the wells with corresponding CD3^+^T cells cultivated alone were subtracted. The values measured in co-cultures of HEp-2 cells and CD3^+^T cells that were primed with control untreated DCs, were taken as 100%.

### Flow cytometry and immunocytochemistry

The analysis of DCs on flow cytometer (Coulter, XL-MCL, Krefeld Germany) or confocal microscope (Zeiss LSM 510/Axiovert 200 M, Jena, Germany) was performed upon labeling the cells with primary antibodies (Abs). The following Abs (clones) and reagents were used for the immunocytochemistry and flow cytometry: IgG1a negative control–biotin (MCA928), anti-CD1a-phycoerythrin (PE) (NA1/34HLK), IgG1 negative control–PE (MCA928PE), anti-CD14-Fluorescein isothiocyanate (FITC) (TUK4), anti-CD86–FITC (BU63), anti-CD3-FITC (UCHT1), IgG1 negative control–FITC (MCA928F) (all from Serotec, Oxford, UK), anti-CD45-PECy5 (HI30), anti-HLA-DR-biotin (LN3), IgG1a negative control-PECy5 (P.3.6.2.8.1) (all from eBioscience), streptavidin-Alexa 488, anti-CD83-Alexa 488 (all from Biolegend). The expression of HLA-DR, CD86 and CD83 was analyzed within CD45^+^ DC population. Necrosis of DCs cultivated with GNPs (5–200 µg/ml) was measured after 48 h-cultures by staining the cells with propidium iodide (PI, 10 µg/ml Sigma) in phosphate buffer saline (PBS). Apoptosis was determined after 48 h by staining the cells with PI in hypotonic citric/Triton-X buffer, or after 24 h by Annexin-V-FITC/PI (R&D) labeling.

### Calcium imaging

Immature DCs (1×10^5^/ml) were cultivated on poly-L-lysine (PLL, 10 µg/ml, Sigma) pre-treated cover-slips, washed with Krebs-Ringer Buffer (KRB), and loaded with Fluo-3 Ca^2+^-indicator (4 µM, Invitrogen) for 30 min at room temperature, followed by washing and incubation at 37°C for 20 min. In some experiments 2 µM thapsigagrin, the inhibitor of sarco/endoplasmic reticulum Ca2+-ATPase (SERCA), in 0.5 mM EGTA, was used during the loading. Cover-slips were then transferred on imaging-chambers in KRB and analyzed on Leica TCS SP5 using a Leica HCX APO L water immersion objective. After initial recording of immature DCs, GNPs (10 µg/ml) and/or LPS were added. The light scattered from GNPs was detected upon excitation at 633 nm by a 660/30 nm Leica HyD hybrid detector (Leica Microsystems GmbH, Wetzlar, Germany). The images were acquired at a frequency of 2 Hz per channel for a total of 5 min. Similar analysis was performed on cells cultivated with LPS (100 ng/ml) and/or GNPs (10 µg/ml) for 24 h and 48 h. The fluorescence signals were expressed as ΔFt/F_0_ ratios, ΔFt representing the fluorescence signal recorded at individual time points, minus F_0_, the initial level of fluorescence. Areas under peaks and frequencies were calculated using Graph Pad Prism software (La Jolla, CA, USA).

### Internalization studies

DCs (1×10^5^/ml) that adhered to the cover-slips were transferred to +4°C and treated with GNPs (10 µg/ml). In some experiments, the dynamin I inhibitor Dynasore (80 µM, Sigma), or DMSO as a vehicle, were added 30 min prior to GNPs. After 20 min, membrane stain FM 4–64 (10 µM, Invitrogen) was applied and the cover-slips were transferred to imaging-chambers and analyzed on a Leica TCS SP5. The cells were monitored for up to 4 h.

Additionally, after the 4 h cultures of DCs with GNPs (10 µg/ml), the cover-slips were washed in Millonig's buffer, fixed in 2% glutharaldehyde overnight at +4°C, and post-fixed with 1% osmium tetroxide for 1 h. After dehydration and resin embedding, ultra thin sections were made and collected on 200-mesh carbon-coated copper grids. The samples were then observed by TEM (JEM100 CX-JEOL, Tokyo, Japan) at 100 kV.

Alternatively, after the osmium tetroxide fixation, another post-fixation with 1% tetracarbohydrazide (TCH) for 30 min and osmium tetroxide for 1 h (OTO staining) was performed for the FIB/SEM analysis. The samples were then dried in serial dilutions of ethanol and hexamethyldistilazane (HMDS), gold sputtered for 3 min at 10 mA and then analyzed on an FIB/SEM FEI Quanta 3D 200 (Oregon, USA). The sample stage was angled to 52°, and the regions of interest were covered with a 1 µm thick Pt layer at the Ga^+2^- sourced FIB current of 1 nA. Regular cross-sections were performed with FIB at 7 nA and 5 µm depth, followed by the cleaning of cross-sections with a current of 300 pA. After each FIB milling/cleaning step, the cells were analyzed with SEM using detectors for secondary (SE) or backscattered electrons (BSE). At least 10 cross-sections/cell, 0.5–1 µm apart were performed during the analysis, on a total of 10 cells/sample.

Quantification of GNPs (10 µg/ml) within DCs after 4 h cultures was determined by Proton-Induced X-ray Emission Spectroscopy (micro-PIXE), as described by Ogrinc et al. [36]. Briefly, DCs were seeded on 1 µm thick ethanol-sterilized gelatin coated Mylar foils. GNP_10_ or GNP_50_ (10 µg/ml) were then added to DCs cultures for 4 h. The samples were washed, plunge-frozen in liquid nitrogen and freeze-dried at −40°C. The mounted samples were analyzed by proton beams (2.5–3 MeV; beam diameter 1.2–2 µm) at different scan sizes. Two PIXE spectra were extracted from a pair of X-ray detectors and chopper, and the elemental mass inventories in single DCs were calculated by using thin sample approximation.

### Cytokines

The levels of cytokines from culture supernatants were quantified by ELISA commercial kits (R&D), according to manufacturer's protocol. The unknown concentrations of cytokines from DCs cultures (IL-12p70, IL-10, IL-23) and DC/CD3^+^T cell co-cultures (IFNγ, IL-4, IL-17) were calculated from standard curves after the subtraction of blank controls.

### Statistical analysis

To evaluate the differences between the experimental and corresponding control samples, the data was analyzed using Friedman's ANOVAs for Ranks, or repeated measures ANOVAs with Bonferroni posttests, if the data followed Gaussian distribution. Kruskal-Wallis or Mann-Whitney tests where used on data from Ca^2+^ oscillation measurements and micro PIXE analysis, respectively. All tests were two-sided and tested at α = 0.05.

## Results

### Cytotoxicity of gold nanoparticles

**Figure 1:**
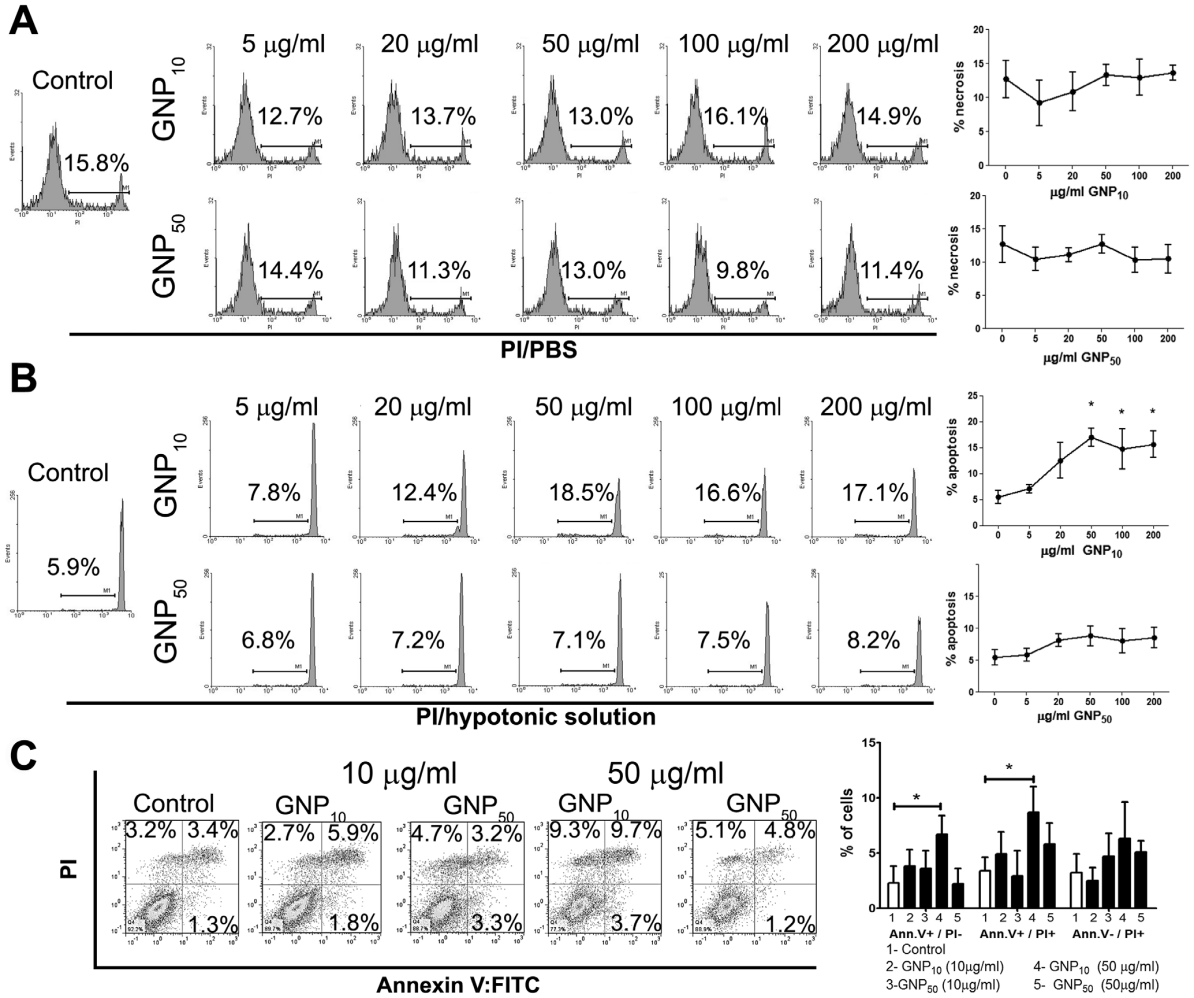
Dose-dependent effect of GNPs on death of DCs. (A) Necrosis and (B) apoptosis of DCs after 48 h cultures is shown from one representative experiment, or as mean ± SD (n = 4 experiments), after PI staining. (C) Different stages of DCs apoptosis were analyzed after 24 h cultures by annexin V:FITC/PI staining, and the results are presented from one representative experiment, or as mean ± SD of three independent experiments. *p<0.05 compared to control (Friedman's one-way ANOVA).

Generation of monocyte-derived DC-based anti-tumor vaccines usually includes a 48 h maturation step with TLR agonists and/or pro-inflammatory cytokines [37]. Therefore, we first investigated whether GNPs (5–200 µg/ml) were cytotoxic for DCs during that period. The examined GNPs did not induce necrosis, even at the highest concentration applied (Figure 1A). However, the apoptosis studies revealed that GNP_10_ increased slightly the percentage of end-stage apoptotic cells (up to 20%) at the concentrations of 50 µg/ml, and the percentage did not increase further with the increase of GNP_10_ concentrations, which pointed to their weak pro-apoptotic effect (Figure 1B). This was confirmed by Annexin-V:FITC/PI staining of DCs cultivated with GNPs (10 µg/ml or 50 µg/ml) for 24 h, where GNP_10_ at a higher concentration (50 µg/ml) increased significantly the percentage of early apoptotic (Annexin-V^+^/PI^−^) and late apoptotic/secondary necrotic cells (Annexin-V^+^/PI^+^), but not primary necrotic cells (Annexin-V^−^/PI^+^) (Figure1C).

### Effects of gold nanoparticles on maturation and functions of dendritic cells

**Figure 2:**
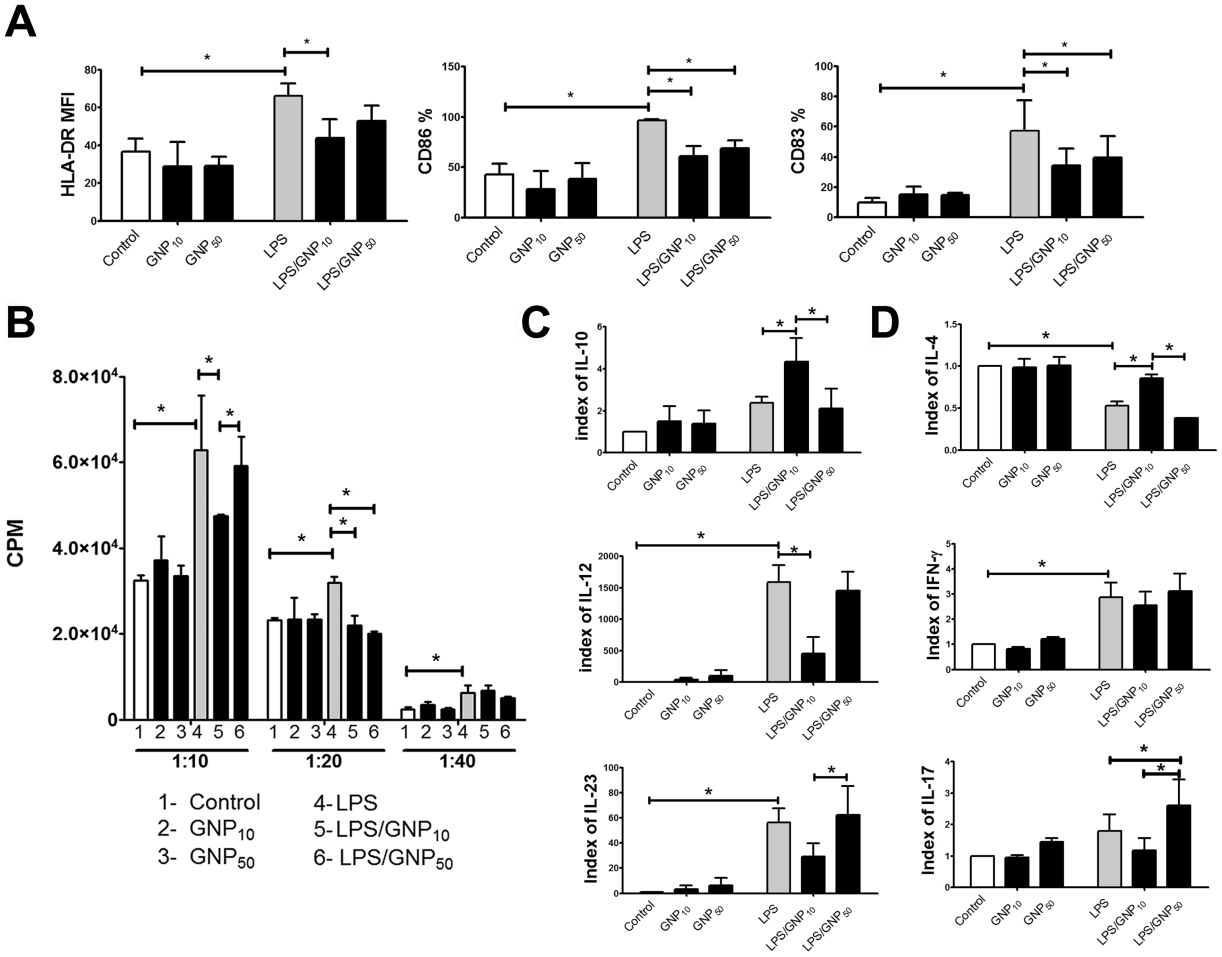
Effect of GNPs on LPS-induced maturation and functions of DCs. (A) Phenotypic maturation of DCs is shown as mean ± SE (n = 4 experiments); see also Figure S1; MFI-mean fluorescence intensity (B) DCs' capacity to stimulate the proliferation of allogeneic CD3^+^T cells, at 3 different DC/CD3^+^T ratios (X-axis), is shown as mean CPM ± SD of six replicates, from one representative experiment. (C) DCs' ability to produce IL-12p70, IL-23 and IL-10 after 48 h; (D) DCs' ability to induce production of cytokines by CD3^+^T cells in co-culture. (C) and (D) are presented as mean indexes ± SD (n = 4 experiments). *p<0.05 (Friedman's two-way ANOVA).

Next, we studied whether GNPs applied at non-cytotoxic concentrations (10 µg/ml), modulate the maturation of DCs induced by LPS. LPS stimulated significantly the expression of HLA-DR, CD86 and CD83 by DCs. Although GNPs alone did not modulate the expression of these molecules, both GNPs impaired significantly the LPS-induced expression of CD86 and CD83 by DCs. Additionally, GNP_10_ suppressed the LPS-induced expression of HLA-DR. (Figure 2A, Figure S1A).

The impaired phenotypic maturation of DCs cultivated with LPS and GNPs correlated with their lower allostimulatory capacity in the co-culture with allogeneic CD3^+^T cells. Thereby, the inhibitory effect of GNP_10_ was stronger and more consistent for different DC-to-CD3^+^T cell ratios in four allogeneic co-cultures performed. A representative allogeneic proliferation is shown in Figure 2B.

The levels of cytokines varied greatly with each donor used in the experiments (Table S1), so the levels of cytokines were analyzed as indexes of control (1.0) (Figure 2C and D). By analyzing the change in production of IL-12p70, IL-23 and IL-10 in DCs culture supernatants we found that GNP_50_ had no effects on their production by DCs. The effects of GNP_10_ differed significantly from that of GNP_50_, since they stimulated significantly IL-10 production by LPS-treated DCs, inhibited significantly the LPS-induced production of IL-12p70, and tended to inhibit the up-regulation of IL-23 (Figure 2C). Consequently, compared to LPS/GNP_50_-treated DCs which up-regulated the production of IL-17 by allogeneic CD3^+^T cells in co-culture, LPS/GNP_10_-treated DCs had significantly higher capacity to induce IL-4. The levels of IFN-γ were not modified in those co-cultures (Figure 2D).

To assess whether LPS was inactivated upon interaction with GNPs, we incubated LPS with GNP_10_ and GNP_50_ for 48 h, as described in Materials and Methods, and afterwards cultivated DCs in the supernatant, or in washed pellet preparations that either contained GNPs or not. We found that the expression of HLA-DR, CD86 and CD83 by DCs (Table S2), and their allostimulatory capacity (data not shown), was not affected significantly by either the supernatant or washed pellet of LPS/GNPs (10 µg/ml) preparations. However, the supernatant activity of the LPS preparation incubated with a higher concentration of GNP_50_ (50 µg/ml) was significantly lower, and the corresponding pellet activity was significantly higher, compared to corresponding controls, as judged by the expression of CD83 by DCs (Table S2). In contrast, the washed pellet preparations with GNPs incubated without LPS had no significant stimulatory effects on the expression of these markers by DCs (data not shown). These results suggested that there was no significant inactivation of LPS, even after potential adsorption to GNPs, and the effects rather occurred via the modulation of signaling mechanisms.

### Effects of gold nanoparticles on Ca2+ oscillations of dendritic cells

**Figure 3:**
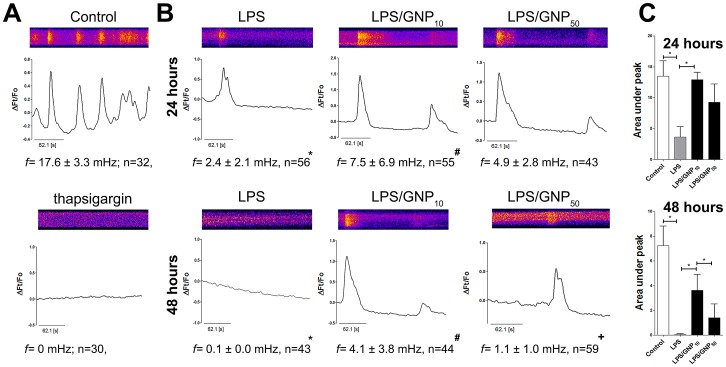
Effect of GNPs on Ca^2+^ oscillations in DCs. Fluo-3 loaded immature DCs were detected (A) immediately upon staining in the presence or absence of thapsigagrin (2 µM), or (B) after 24 h or 48 h in the presence of LPS and GNPs (10 µg/ml), as indicated. Representative kymographs and corresponding ΔFt/F_0_ records are shown. The frequency of oscillations and, (C) area under peaks, are presented as mean ± SD of all analyzed cells. See also Figure S2. *p<0.05, compared to control or as indicated; ^#^p<0.05 compared to LPS; ^+^p<0.05 compared to GNP_10_ (Kruskal-Wallis test).

Vukcevic et al. [8] showed that LPS down-regulates spontaneous Ca^2+^ oscillations in DCs and transfer of NFAT to their nucleus. Accordingly, we investigated whether GNPs impair the Ca^2+^ signaling in DCs during maturation. Fluo-4-loaded immature DCs displayed high Ca^2+^ fluctuations, corresponding to the average area under peaks of 18.6±6.2, which could be blocked completely by thapsigagrin (Figure 3A, Figure S2). Immediate changes in Ca^2+^ oscillation were not detected upon addition of LPS (data not shown) or GNPs (Videos S1–S3). Ca^2+^ fluctuations in control DCs declined progressively during 48 h cultivation (Figure S2), but LPS down-regulated significantly the Ca^2+^ fluctuations after both 24 h and 48 h (Figure 3B, 3C, Videos S4–S6). However, if DCs were treated with LPS/GNP_10_, significant inhibition of such down-regulation was observed (Figure 3B and C). After 48 h, DCs treated with LPS/GNP_10_ possessed significantly higher Ca^2+^ oscillations compared to those in LPS/GNP_50_ treated DCs (Figure 3B). Such effects of GNPs were not observed in the absence of LPS (Figure S2).

### Internalization of gold nanoparticles

**Figure 4:**
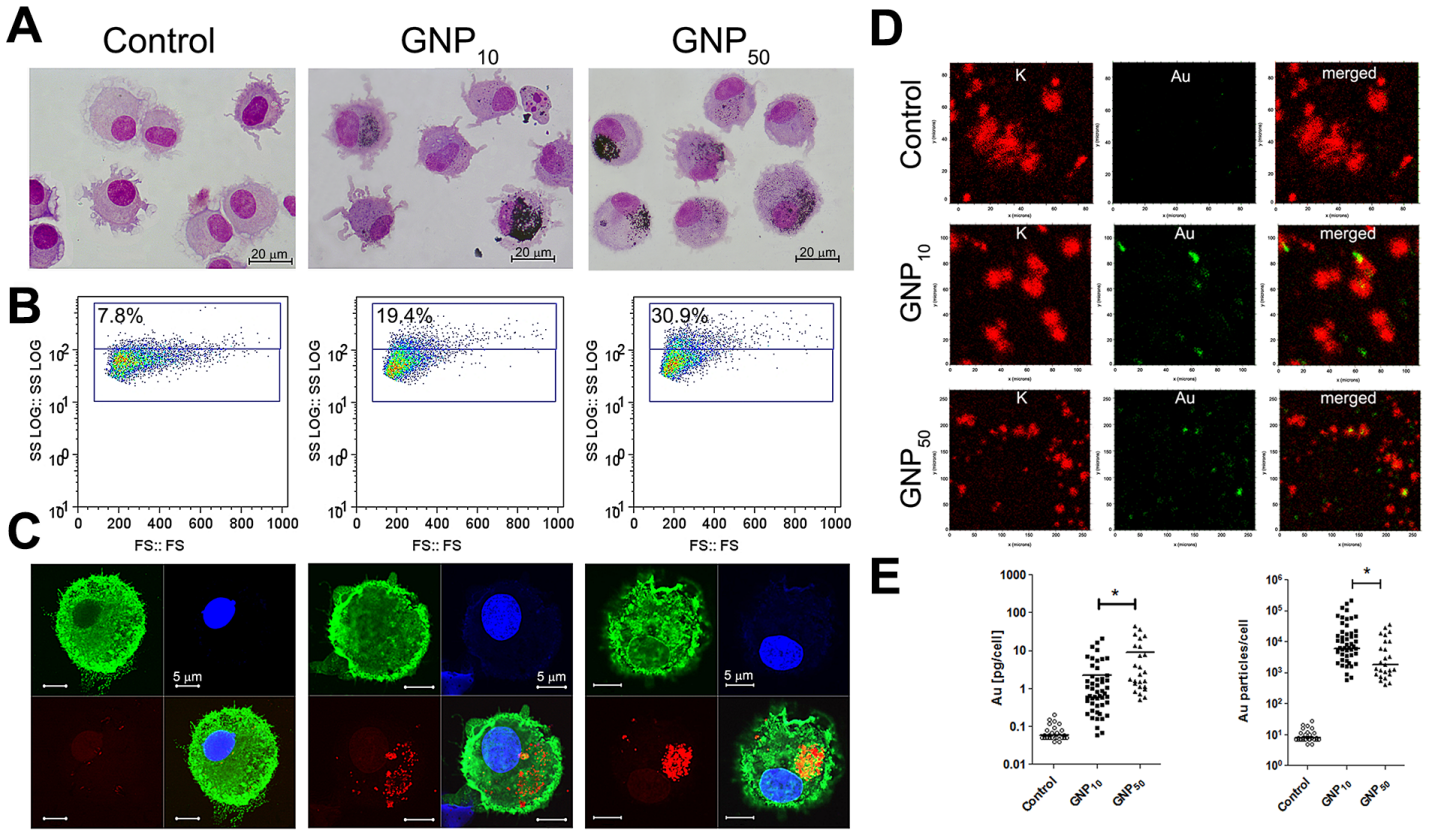
Internalization of GNPs by DCs. Intracellular GNPs were analyzed in 48-cultures (A) after staining with MGG, (B) by flow cytometry, or (C) confocal microscopy. (D) Micro-PIXE analysis was used for the quantification of potassium (red) and gold (green) after 4 h cultures. Representative elemental maps are shown. (E) All data from micro-PIXE analysis is presented either as the amount of gold [pg/ml] per cell, or as the number of GNPs per cell, calculated from GNPs' size and density of Au (ρ = 19.3 g/cm^3^). *p<0.05 (Mann-Whitney test).

Next, we investigated how the size-dependent differences in the immunomodulatory properties of GNPs correlate with their internalization by DCs. Light microscopy analysis showed a great variability in the internalization capacity of DCs. Still, a significantly higher percentage of DCs internalized GNP_50_ (82.1%±4.3%; n = 500), compared to GNP_10_ (68.5%±3.1%; n = 500) (p = 0.013) (Figure 4A). Similar results were obtained when analyzing DCs' internal complexity as a side scattering parameter on the flow cytometer (Figure 4B). The intracellular distribution of GNPs analyzed by confocal microscopy showed that GNPs had exclusively perinuclear localization (Videos S7 and S8), but GNP_50_ appeared more clustered, and GNP_10_ more dispersed (Figure 4C).

To quantify the intracellular GNPs, we applied the micro-PIXE analysis [36] (Figure S3). The cells were identified by potassium elemental maps, so the amount of gold was determined within these areas (Figure 4D). No correlation was observed between the potassium area size, corresponding to DCs size, and the amount of intracellular gold (Figure S3). Although the amount of intracellular gold varied greatly, we detected a significantly higher average mass of GNP_50_ within DCs, compared to GNP_10_ (Figure 4E). However, when the mass of gold was recalculated to the number of GNPs per cell, a significantly higher number of GNP_10_ per cell was observed, compared to GNP_50_ (Figure 4E).

### Intracellular trafficking of gold nanoparticles

**Figure 5:**
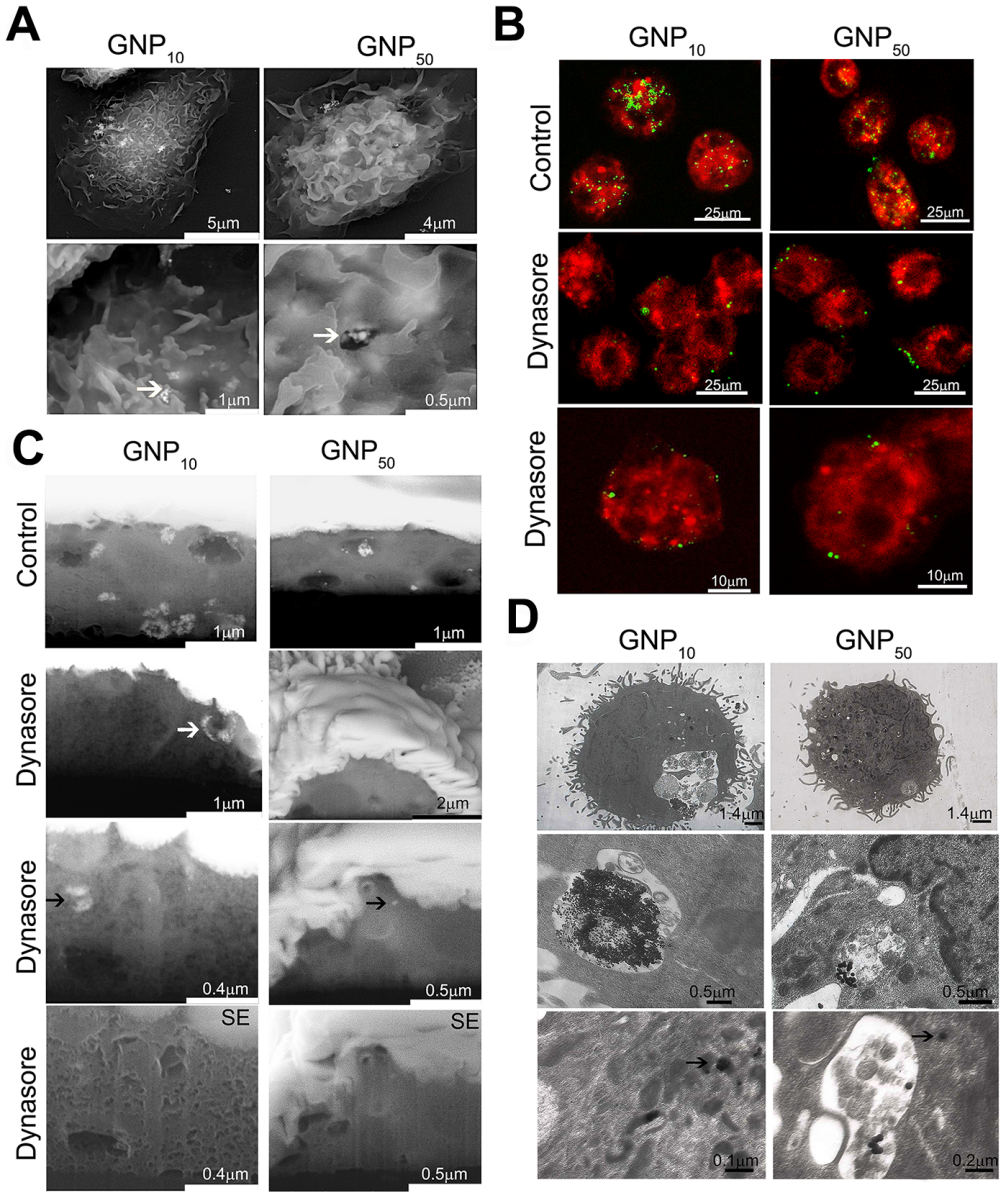
Mechanisms of GNPs internalization by DCs. The internalization was analyzed by (A) SEM, under different magnifications; white arrows point to GNPs-membrane interactions; (B) Confocal microscopy of live cells stained with FM4-64; or (C) FIB/SEM using BSE, or SE detectors where indicated. White arrow points to the GNP_10_-loaded vesicle attached to the outer membrane, whereas black arrows point to intracellular presence of GNP_10_ agglomerates and a single particle of GNP_50_. The cells shown in (B) and (C) were cultivated with GNPs for 3 h in the presence or absence of Dynasore; (D) TEM, after 24 h-cultures under different magnifications. Back arrows indicate intracellular GNP_10_ agglomerates and a single GNP_50_ particle outside the endosome.

In addition to the quantitative differences, we explored the differences in the mechanisms of GNPs' internalization and intracellular distribution. SEM analysis suggested that both GNPs are captured as small clusters by DCs' filopodia predominantly, followed by the membrane enclosing, which is a characteristic for phagocytosis and macropinocytosis (Figure 5A). Afterwards, GNPs were located in the endosomes. No such processes occurred if DCs were cultivated with GNPs at +4°C (data not shown). The Dynasore treatment almost completely blocked the internalization of both GNPs, according to the confocal microscopy (Figure 5B) and FIB/SEM (Figure 5C). However, cross-sectioning of the whole cells with FIB suggested that Dynasore-treated DCs contained more agglomerates of GNP_10_ intracellularly, and only a few nanoparticles of GNP_50_ (Figure 5C). BSE signals, unlike SE signals, were not completely clear due to low spatial resolution of this detector, but the gold composition of the bright intracellular fields was confirmed by EDX detector (data not shown). In addition to FIB/SEM, TEM analysis suggested also that some nanoparticles could reside outside the endosomes (Figure 4D). GNPs were found inside the cytoplasmic tubular membranous structures, either as small aggregates (GNP_10_), or as single particles (GNP_50_).

### Effects of gold nanoparticles on DCs'maturation induced by heat-killed necrotic HEp-2 cells

By interfering with DCs maturation and signaling, GNPs could potentially induce adverse effects when used in photo-thermal therapy. To evaluate this hypothesis, we cultivated HEp-2 cells with GNPs for 24 h, killed them by heat, and treated DCs with the necrotic HEp-2 cells, after which GNPs were found inside the DCs (Figure S4). The induction of necrosis by heat was chosen since the laser ablation of control HEp-2 cells was not as efficient as for those which internalized GNPs (data not shown) [35].

**Figure 6:**
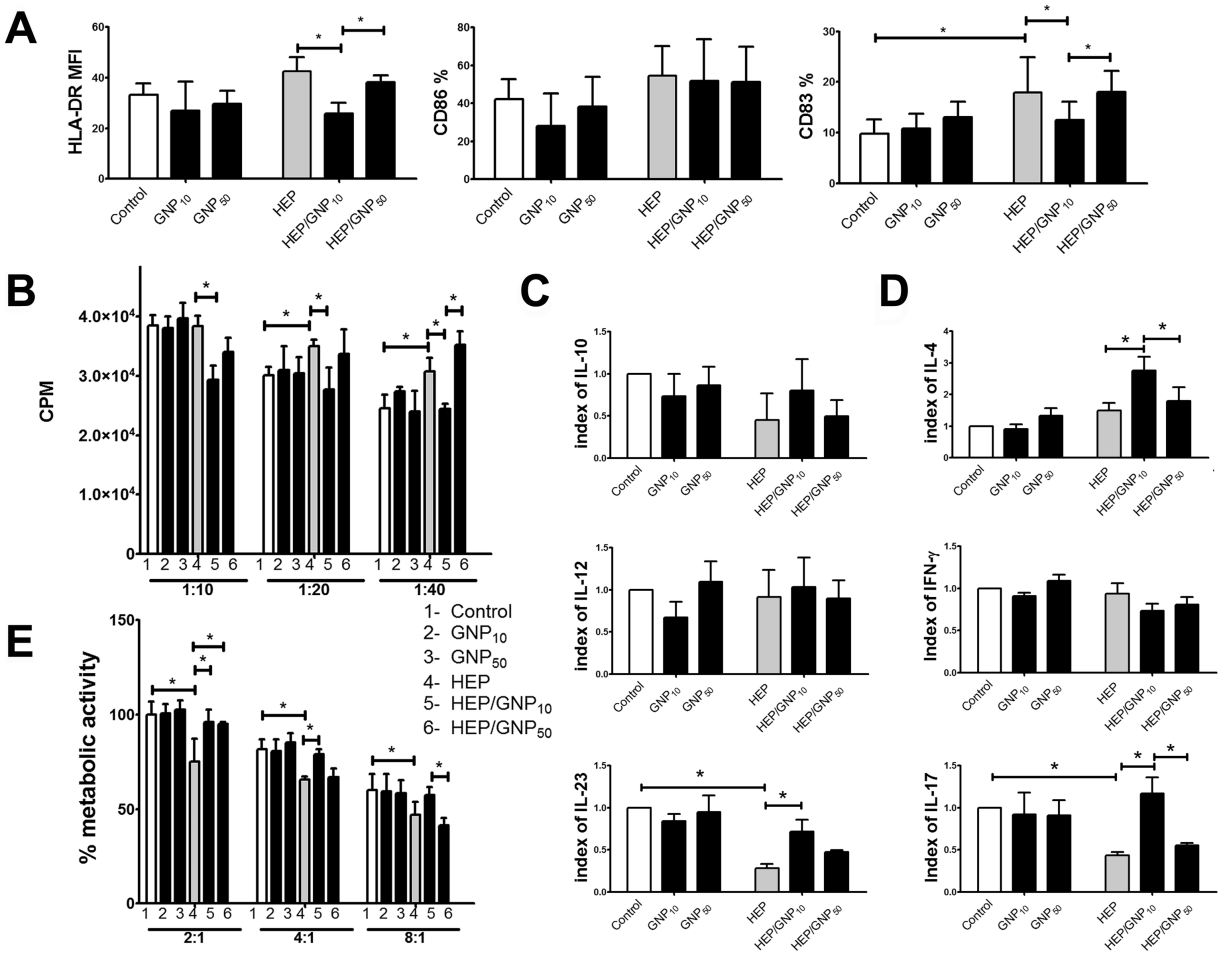
Effect of GNPs on necrotic HEp-2-induced maturation and functions of DCs. (A) Phenotypic maturation of DCs is shown, as mean ± SE (n = 4 experiments); see also Figure S1; MFI-mean fluorescence intensity (B) DCs' capacity to stimulate the proliferation of allogeneic CD3^+^T cells, at 3 different DC/CD3^+^T ratios (X-axis), is shown as mean CPM ± SD of six replicates, from one representative experiment. (C) DCs' ability to produce IL-12p70, IL-23 and IL-10 after 48 h; (D) DCs' ability to induce production of cytokines by CD3^+^T cells in co-culture. (C) and (D) are presented as mean indexes ± SD (n = 4 experiments). (E) Metabolic activity of live HEp-2 cells co-cultivated with CD3^+^T cells, previously primed with DCs for 5 d. Results from a representative MTT assay are shown as mean ± SD of six replicates *p<0.05 (Friedman's two-way ANOVA).

Necrotic HEp-2 cells induced only slightly higher HLA-DR and CD86 expression, but significantly higher CD83 expression on DCs (Figure 6). However, if the necrotic HEp-2 cells were previously cultivated with GNP_10_, DCs possessed significantly lower expression of HLA-DR and CD83, compared to both HEp-2-treated and HEp-2/GNP_50_-treated DCs.

Impaired maturation of DCs cultivated with necrotic HEp-2 cells and GNP_10_, but not GNP_50_, was followed by their diminished allostimulatory capacity in at least two different DC-to-CD3^+^T cell ratio per allogeneic co-culture of totally four performed (Figure 6B).

Control necrotic HEp-2 cells, and those loaded with GNP_50_, lowered significantly the IL-17-inducing capacity of DCs, which correlated with the down-regulation of IL-23 production by DCs. Such an effect on IL-17 was inhibited by GNP_10_ (Figure 6C, Table S1). GNP_10_ also enhanced the ability of HEp-2-treated DCs to stimulate the production of IL-4 by CD3^+^T cells (Figure 6D), suggesting that HEp-2/GNP_10_-treated DCs could have an impaired anti-tumor activity.

To investigate this hypothesis, CD3^+^T cells primed with DCs, were cultivated with live HEp-2 cells, as described. Indeed, CD3^+^T cells primed with DCs that were matured with necrotic HEp-2/GNP_10_, had significantly lower cytotoxic activity compared to CD3^+^T cells primed with control HEp-2-treated DCs. The differences between GNP_10_ and GNP_50_ in such an effect were more significant at higher CD3^+^T/HEp-2 cell ratios. All these effects of GNPs were observed only in the presence of necrotic HEp-2 cells (Figure 6E).

## Discussion

Many papers showing that GNPs accumulate in tumor cells, suggested enthusiastically that GNPs could be used as tumor-contrasting agents and in photo-thermal cancer therapy [14], [17]. However, it remained unclear what could be the consequences of such application for the immune system, a crucial part of the successful anti-tumor therapy [1]. The immunosuppressive effects of GNPs have been suggested as potentially adverse in cancer therapy [11], [27], but no one investigated this issue directly. To address this, we investigated the effects of GNPs on DCs, the key cells involved in the regulation of anti-tumor response [4]. The higher concentrations of GNP_10_ (50 µg/ml or higher) had a weak pro-apoptotic effect, since up to 20% of cells died by apoptosis in 24 h and 48 h cultures. We did not observe a further increase of apoptosis with higher concentrations of GNP_10_ (100 and 200 µg/ml), probably because the maximal phagocytic capacity of DCs was already reached at the lower concentrations of GNP_10_. The cytotoxicity of GNPs has been described previously [26], [38], and the effect was stronger compared to our results. A similar reduction of viability of Hep-G2 cells (up to 20%) was observed after the treatment with 10 nm GNPs (100 µM, corresponding to 2*10^12^ GNPs/ml), and the effect was described as non-cytotoxic [39]. The weak pro-apoptotic effect of GNP_10_ in our experiments was observed at much higher concentrations, (50 µg/ml corresponding to 5*10^12^ particles/ml and higher). These results are in accordance with the conclusion of Khlebsov and Dykman [40], that GNPs at concentrations up to 1*10^12^/ml are non-cytotoxic for various cells. In line with this, GNP_50_ had no cytotoxic effects on DCs up to 200 µg/ml (corresponding to 0.2 * 10^12^/ml), similarly to GNP_10_ at a concentration of 10 µg/ml (corresponding to 1.1 * 10^12^). The described mechanisms of GNPs cytotoxicity included the alterations of proteins functions [41], or disruption of endosomal membrane [42]. Interestingly, Villiers et al. [27] did not observe the cytotoxic effect of 10 nm-sized GNPs on immature DCs in a single evaluated dose (0.5 mM), which could be due to the different maturation state of DCs, or different capping agent used for GNPs' stabilization in their study.

The key findings in our study were that the non-toxic concentrations of GNP_10_ can impair the maturation and functions of DCs, along with their capacity to induce T-cell mediated anti-tumor response. In line with Villiers et al. [27], the effects of GNPs were significant only in the presence of maturation stimuli. For the first time, the effects of GNPs were explored on the model of LPS-induced maturation of human DCs and the maturation induced by necrotic tumor cells generated at conditions similar to those induced in GNP-based photo-thermal therapy [35], both of which activate a TLR4 signaling pathway [2], [3], [6]. Recently, Tsai et al. [28] showed that GNPs could suppress CpG-induced, but not LPS-induced, maturation of macrophages by inhibiting the translocation of TLR9 to phagosomes via HMBG-1-dependent mechanisms. In contrast, we showed that GNPs, especially the smaller ones, inhibited the LPS-induced maturation and functions of DCs, and the effect did not occur due to LPS inactivation by GNPs, but rather by the modulation of cellular signaling. Such disagreements could be expected since macrophages and DCs respond differently to LPS [43]. Here, we showed that GNPs impaired the up-regulation of CD83 by DCs, which is crucial for the stability of their maturation in an IL-10-enriched immunosuppressive tumor micro-environment [44]. A significant down-regulation of LPS-induced HLA-DR expression probably resulted in more consistent impairment of allostimulatory capacity of these DCs, since HLA-DR is one of the two signals necessary for CD3^+^T cells' proliferation. The lower proliferative capacity of LPS/GNP_10_-treated DCs could be a consequence of increased production of IL-10 immunoregulatory cytokine, which is also involved in the promotion of Th2 development [5]. In line with this, Th2 cells were shown rather to promote tumor growth, unlike Th1 cells specific for the same antigen [45]. In addition, GNP_10_ impaired the LPS-induced up-regulation of IL-12p70, an Th1-inducing cytokine with anti-tumor effects [7]. In line with our results, Villers et al. [27] showed that 10 nm-sized GNPs can suppress LPS-induced up-regulation of IL-12 production by mouse DCs. However, the lower production of IL-12p70 by LPS/GNP_10_-treated DCs was not followed by lower production of IFN-γ by CD3^+^T cells, suggesting that additional cytokines might be involved in its expression [7]. Unlike GNP_50_ which stimulated Th17 development, GNP_10_ tended to down-regulate it. The presence of Th17 cells in tumor tissues was described either as antitumoral or protumoral, but simultaneous induction of Th17 and Th1 responses are described often as desirable in tumor therapy [46]. Therefore, the overall effect of GNP_10_ on Th development, unlike that of GNP_50_, could be interpreted as adverse in the tumor therapy.

Even when DCs maturation was induced by necrotic tumor cells, GNP_10_ were able to suppress the maturation and allostimulatory capacity of DCs. Furthermore, GNP_10_, unlike GNP_50_, promoted the Th2/Th17 polarization capacity of DCs. Considering the protumoral effects of Th2 cells [45], and the fact that IL-17 alone can support tumor growth and metastasis [46], this experimental model also confirmed that GNP_10_ could potentially have adverse immunomodulatory effects if used in photo-thermal tumor therapy. Indeed, we observed an impaired cytotoxic activity of CD3^+^T cells primed with DCs that were matured with GNP_10_-loaded necrotic HEp-2 cells. Although the response of DCs to heat-killed necrotic tumor cells was shown to be dependent on TLR4 expression [34], we observed that the effects on Th polarization are different from that of LPS. In line with this, Kandil et al. [34] suggested that, unlike LPS, heat-killed necrotic tumor cells can induce the production of IL-12p40 by DCs, but not the production of IL-12p70, a bioactive form of this cytokine. So, the different effects of necrotic cells on DCs maturation could be due to activation of additional signaling pathways. For example, both hsp60 and HMGB1 were shown to activate TLR2 [47], [48], in addition to TLR4 [2]. The inhibitory role of TLR2-dependent SOCS-1 activation on TLR-4- and TLR7/8-induced up-regulation of IL-12 and IL-23 production has been described as an important immune checkpoint suppressing the overwhelming inflammation [49]. The co-ligation of TLR2 and TLR4 on DCs was recently shown to potentiate their immunogenicity toward a tumor [50], and our results suggest that this process could be inhibited by GNP_10_. These results, in line with our previous findings showing that the smaller GNPs potentiate production of IL-10, unlike the larger ones [31], suggest that the phenomenon of GNPs-mediated immunosuppression does not depend on the experimental model applied or the method of GNPs preparation.

The size-dependent immunomodulatory effects of GNPs could be attributed to different mechanisms of their internalization, levels of accumulation and intracellular distribution within DCs, leading to different modulation of maturational signaling. Upon interaction with the medium, various proteins and ions, including LPS, adsorb to the negatively charged surface of GNPs, as judged by the increase of GNPs' hydrodynamic size, polydispersion index (PDI) and decrease of zeta-potential in complete medium (Table 2), which facilitates their uptake by DCs, and activates downstream signaling pathways [51]. Our data is in agreement with other studies demonstrating that the uptake of GNPs is an energy dependent process occurring via endocytosis [20]. We showed that the process is predominantly dynamin-dependent, but also that GNP_10_ were better in entering DCs via dynamin-independent mechanisms. The latter mechanisms seem to be more prominent in non-phagocytic cell lines, where Dynasore could reduce the internalization of GNPs by only 42% [23]. The alternative routes for GNPs internalization could be mediated by clathrin- and dynamin-independent carriers (CLICs), which are regulated by CDC42 protein [22]. The tubular membranous structures, 50–80 nm wide, that we observed by TEM resembled CLICs. The CLIC-like structures could have acted as size-selective filters for the preferential internalization of GNP_10_ during dynamin blockage experiments. In support of this hypothesis, we observed that the tubular structures contained clusters of GNP_10_, but only 1–2 GNP_50_. CDC42 is involved in the regulation of macropinocytosis, phagocytosis and other signaling pathways in DCs [52]. Therefore, GNP_10_ could have affected different signaling processes involved in the maturation of DCs, by utilizing additional routes of internalization.

GNPs were found predominantly within endosomes/lysosomes upon internalization, which is in line with the other reports [20], [21], [23]. Such localization could improve the GNPs-mediated delivery of endosomal TLR agonists into DCs, such as CpG [11] or 7-thia-8-oxoguanosine [53]. However, we also found GNPs outside the endosomes, especially the smaller ones. Endosomal escape of GNPs could be expected for tumor cells [23], but has never been described for DCs. Such an effect of GNPs could be beneficial if used for delivery of specific agonists to cytoplasmic receptors in DCs, such as MDA-5 and NOD-like receptors, thereby promoting their maturation and functions [6]. The endosomal escape of GNPs could even increase if such functionalization produces a positive surface charge enabling the “proton sponge” effect [54]. Since our GNPs were negatively charged, with similar z-potential in cell culture medium (Table 2), the mechanism of endosomal escape via a “proton sponge” effect is highly unlikely, and rather occurred accidentally via exposure to light [24]. Within endo/lysosomes and cytoplasm, GNPs were shown to modify the activity of different proteins, such as cathepsins [55] or HMBG-1 [28], respectively, which are important regulators of DCs' maturation and functions [56], [57]. Therefore, GNP_10_ has probably modified more different proteins compared to GNP_50_, by utilizing additional routes of internalization and by endosomal escape. Even though the light microscopy and flow cytometry suggested different results, measurements on micro-PIXE showed that the number of GNPs per cell, and not their intracellular mass or the percentage of cells which internalized GNPs, could actually be a key factor determining their immunomodulatory effects. Micro-PIXE analysis, applied for the first time in such analysis, was proved as an accurate method for the quantification of GNPs within cells, as well as the variability of this process. In contrast to others [23], we did not observe a significant correlation between the cell size and the amount of gold within the cells. Such variability could rather be explained by the heterogeneity of DCs in phenotypic and maturation state, where the phagocytic activity correlates inversely with the level of their maturation [5].

The role of Ca^2+^ signaling in DCs' maturation has been studied many times, and we showed, for the first time, that GNP_10_, unlike GNP_50_, inhibits significantly the changes in Ca^2+^ oscillations during LPS-induced maturation. Vukcevic et al. [8] showed that the down-regulation of Ca^2+^ fluctuations leads to the cytoplasmic localization of NFAT. In line with their observations, we did not observe immediate changes in Ca^2+^ influx upon stimulation by LPS. Ca^2+^ oscillations were found to be dependent on phospholipase C activity, SERCA, and endoplasmic reticulum's inositol-3-phosphate receptor, but the role of Ca^2+^ influx has also been described [8]. Although it was shown that GNPs could alter the expression of proteins involved in Ca^2+^ oscillations [58], we could not detect a direct effect of GNPs on Ca oscillations in the absence of maturation stimuli. Furthermore, the experiments with blocking of Ca^2+^ oscillations in DCs showed that the oscillations alone are not the sole regulator of nuclear translocation of NF-κB and DCs' maturation [8]. These results suggest that the mechanisms by which GNPs modulate maturation of DCs are more complex. Ca^2+^ fluctuations, in this context, could be a central regulatory factor synchronizing DCs' maturation, which can sense the various signals within the cells, some of which are modulated by the internalized GNP_10_.

In conclusion, our results showed that smaller GNP_10_ have stronger inhibitory effects on maturation and antitumor functions of DCs, induced either by LPS or heat-killed tumor necrotic cells, compared to larger GNP_50_. The molecular mechanisms, by which GNPs act on these processes, probably depend on the levels of particles' accumulation and distribution within DCs, which affects calcium-depended signaling differently. Consequently, the maturation of DCs induced by irradiation of a tumor could be compromised by smaller GNPs, leading to a poor T-cell mediated anti-tumor response. Cumulatively, these results point to potential adverse effects of smaller GNPs, if used in photo-thermal therapy and cancer diagnostics.

## Supporting Information

Figure S1 **Effect of GNPs on phenotypic maturation of DCs.** Representative flow cytometry data on the effect of GNPs is shown, on the expression of HLA-DR, CD86 and CD83 by DC during maturation induced with (A) LPS, or (B) necrotic HEp-2 cells. The marker showing specific fluorescence was adjusted in each experiment (n = 4 per stimuli type) according to fluorochrome-labeled isotype control Abs. M-Mean fluorescence intensity.
(TIF)

Figure S2 **Effect of GNPs on Ca^2+^ oscillations in DCs.** Ca^2+^ oscillations in Fluo-3 loaded immature DCs were detected immediately upon staining (0 h), after 24 h, or 48 h in the presence or absence GNPs (10 µg/ml), as indicated. The oscillations were expressed as area under peaks, and presented as mean ± SD of all analyzed cells. *p<0.05 (Friedman's one way ANOVA).
(TIF)

Figure S3 **Micro-PIXE quantitative analysis of intracellular GNPs.** DCs were cultivated with GNPs on Mylar foils for 4 h and observed by (A) phase contrast microscopy. (B) After plunge freezing and cryo-drying the samples were mounted in a vacuum chamber and observed by CCD camera. (C) The elemental maps were recorded with micro-PIXE on the indicated places of analysis, as described in Experimental details. (D) The cells of interest on the maps were marked by ellipses, and the cells' size was correlated against the amount of potassium or chlorine. Since only the former correlated with area size, potassium maps were taken as markers for cells. The amount of gold was correlated against areas size to observe whether the cell size affected the level of GNP uptake. Correlation analyses were performed in Graph Pad Prism software.
(TIF)

Figure S4 **Co-cultures of GNP-loaded necrotic HEp-2 cells and DCs.** HEp-2 cells were cultivated for 24 h with GNPs (10 µg/ml), harvested and analyzed after staining with MGG by light microscopy (A, E and I). The cells were then heat killed, as described in Methods, and analyzed by light microscopy after staining with Trypan blue solution (B, F and J). The necrotic tumor cells were co-cultivated with immature DCs for 48 h, followed by cell harvesting and preparation of cytospins. The samples were then stained with MGG (C, G and K), or with HLA-DR: Alexa-488 and PI and analyzed by confocal microscopy (D, H and L). GNPs in those experiments were detected by strong light scattering properties wtih 660/30 nm detector.
(TIF)

Table S1 **Cytokines production by LPS-treated DCs (IL-10, IL-12 and IL-23), and by CD4+T cells (IL-4, IFN-γ, IL-17) in subsequent co-culture.** Summarized results are presented as median (range) of all experiments performed.
(DOC)

Table S2 **Interference of GNPs with the effect of LPS on phenotypic maturation of DCs.** GNP_10_ and GNP_50_ (10 or 50 µg/ml) were incubated with LPS (100 ng/ml) in complete RPMI medium for 48 h, after which the supernatant was isolated by centrifugation, and the pellet was washed two times in complete medium. DCs were cultivated in the supernatant or in washed pellet preparation for 48 h, and the expression of indicated markers was measured by flow cytometry. Results are shown as mean ± SD of two independent experiments. *p<0.05 compared to control.
(DOCX)

Video S1 **Ca^2+^ imaging in immature DCs.** The imaging was performed immediately after staining with Fluo-3.
(MOV)

Video S2 **Immediate effect of GNP_10_ on Ca^2+^ oscillations.** Ca^2+^ imaging of Fluo-3 stained immature DCs was performed 10 minutes after addition of GNP_10_ (10 µg/ml).
(MOV)

Video S3 **Immediate effect of GNP_50_ on Ca^2+^ oscillations.** Ca^2+^ imaging of Fluo-3 stained immature DCs was performed 10 minutes after addition of GNP_50_ (10 µg/ml).
(MOV)

Video S4 **Ca^2+^ imaging in LPS-matured DCs after 24 h.** Ca^2+^ imaging of Fluo-3 stained DCs cultivated for 24 h in the presence of LPS in shown.
(MOV)

Video S5 **Effect of GNP_10_ on Ca^2+^ oscillations after 24 h.** Ca^2+^ imaging of Fluo-3 stained DCs cultivated for 24 h in the presence of LPS and GNP_10_ (10 µg/ml) is shown.
(MOV)

Video S6 **Effect of GNP_50_ on Ca^2+^ oscillations after 24 h.** Ca^2+^ imaging of Fluo-3 stained DCs cultivated for 24 h in the presence of LPS and GNP_50_ (10 µg/ml) is shown.
(MOV)

Video S7 **Perinuclear localization of GNP_10_.** Animated 3D scan was obtained by confocal microscopy of DCs cultivated with GNP_10_ (10 µg/ml) for 24 h and stained with HLA-DR:Alexa 488 and PI.
(MOV)

Video S8 **Perinuclear localization of GNP_50_.** Animated 3D scan obtained by confocal microscopy of DCs cultivated with GNP_50_ (10 µg/ml) for 24 h and stained with HLA-DR:Alexa 488 and PI.
(MOV)
