# Supplementary figures and images for: Size-Dependent Effects of Gold Nanoparticles Uptake on Maturation and Antitumor Functions of Human Dendritic Cells In Vitro
Source: PLoS One. 2014 May 6;9(5):e96584. doi: 10.1371/journal.pone.0096584 (PMC4011871; doi:10.1371/journal.pone.0096584)

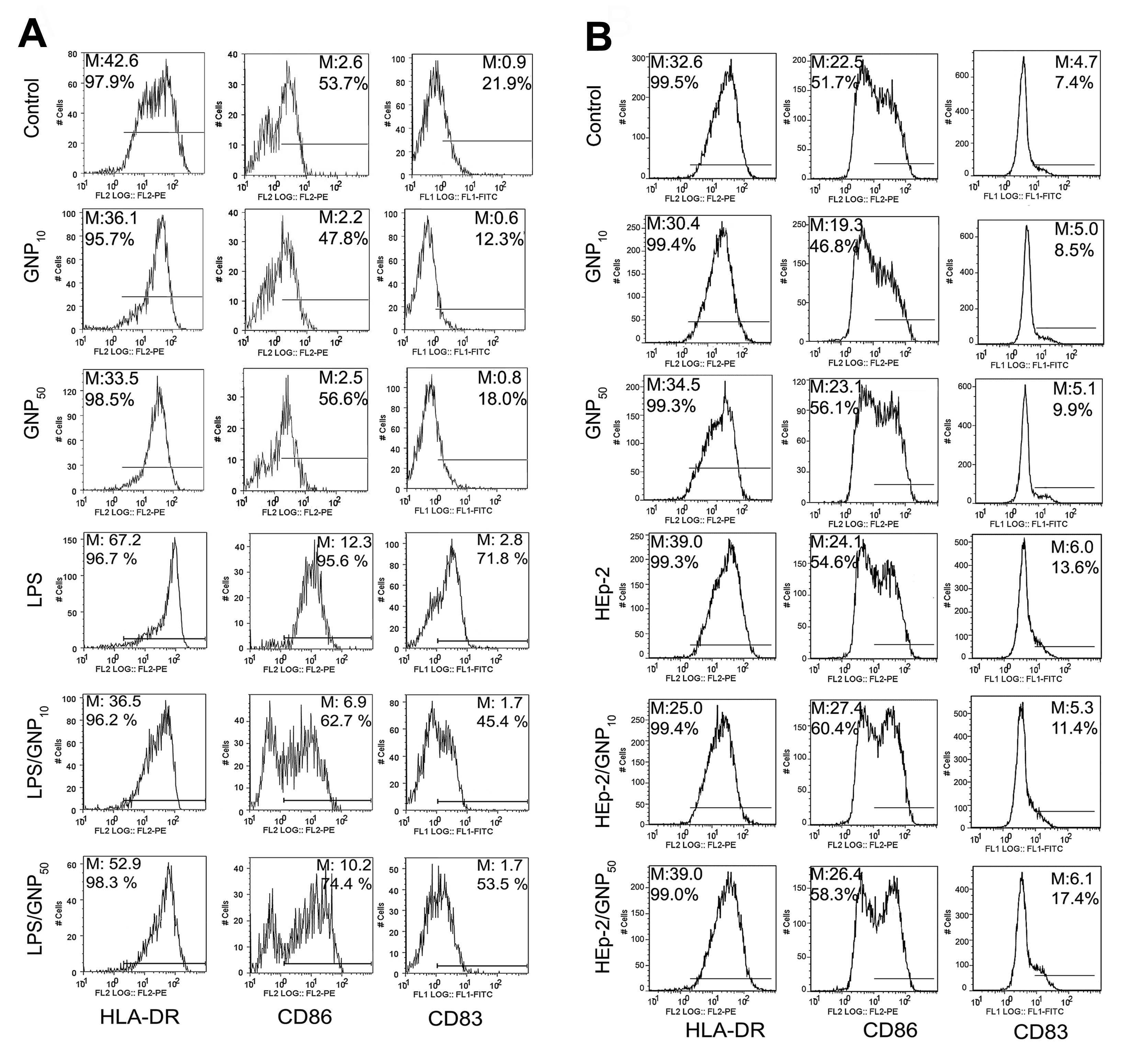

Supplement: Figure S1 — Effect of GNPs on phenotypic maturation of DCs. Representative flow cytometry data on the effect of GNPs is shown, on the expression of HLA-DR, CD86 and CD83 by DC during maturation induced with (A) LPS, or (B) necrotic HEp-2 cells. The marker showing specific fluorescence was adjusted in each experiment (n = 4 per stimuli type) according to fluorochrome-labeled isotype control Abs. M-Mean fluorescence intensity. (TIF) [file pone.0096584.s001.tif]

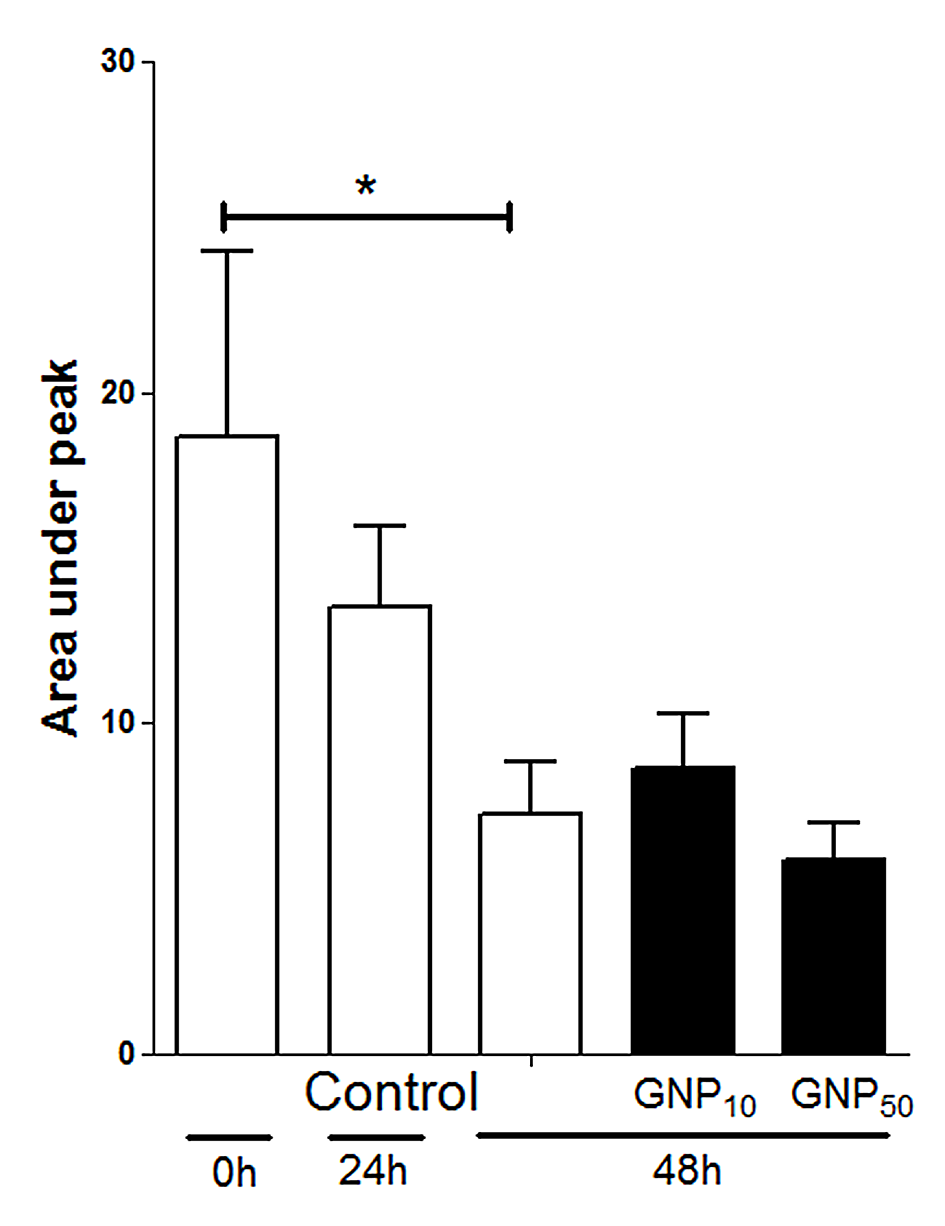

Supplement: Figure S2 — Effect of GNPs on Ca2+ oscillations in DCs. Ca2+ oscillations in Fluo-3 loaded immature DCs were detected immediately upon staining (0 h), after 24 h, or 48 h in the presence or absence GNPs (10 µg/ml), as indicated. The oscillations were expressed as area under peaks, and presented as mean ± SD of all analyzed cells. *p<0.05 (Friedman's one way ANOVA). (TIF) [file pone.0096584.s002.tif]

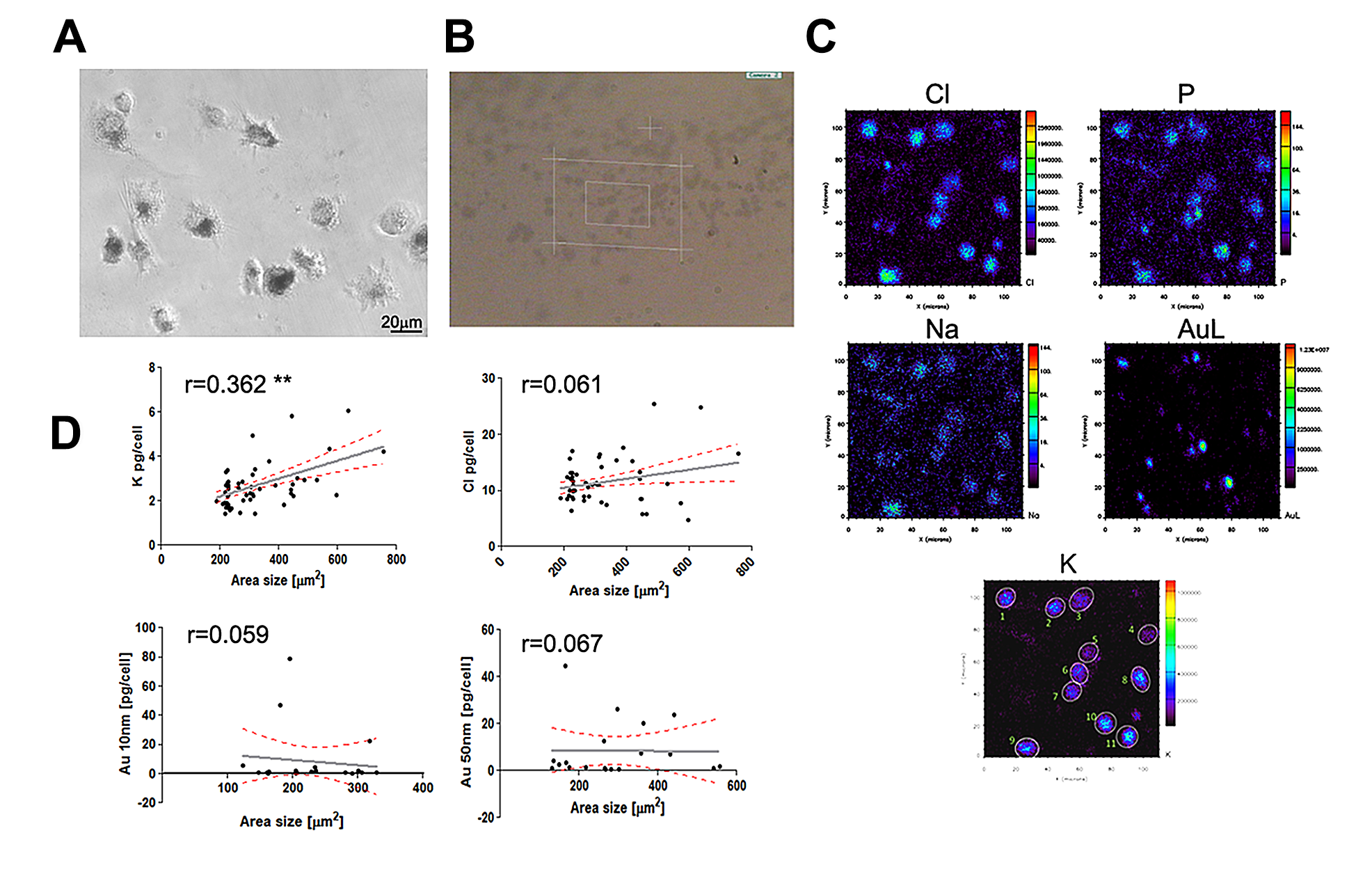

Supplement: Figure S3 — Micro-PIXE quantitative analysis of intracellular GNPs. DCs were cultivated with GNPs on Mylar foils for 4 h and observed by (A) phase contrast microscopy. (B) After plunge freezing and cryo-drying the samples were mounted in a vacuum chamber and observed by CCD camera. (C) The elemental maps were recorded with micro-PIXE on the indicated places of analysis, as described in Experimental details. (D) The cells of interest on the maps were marked by ellipses, and the cells' size was correlated against the amount of potassium or chlorine. Since only the former correlated with area size, potassium maps were taken as markers for cells. The amount of gold was correlated against areas size to observe whether the cell size affected the level of GNP uptake. Correlation analyses were performed in Graph Pad Prism software. (TIF) [file pone.0096584.s003.tif]

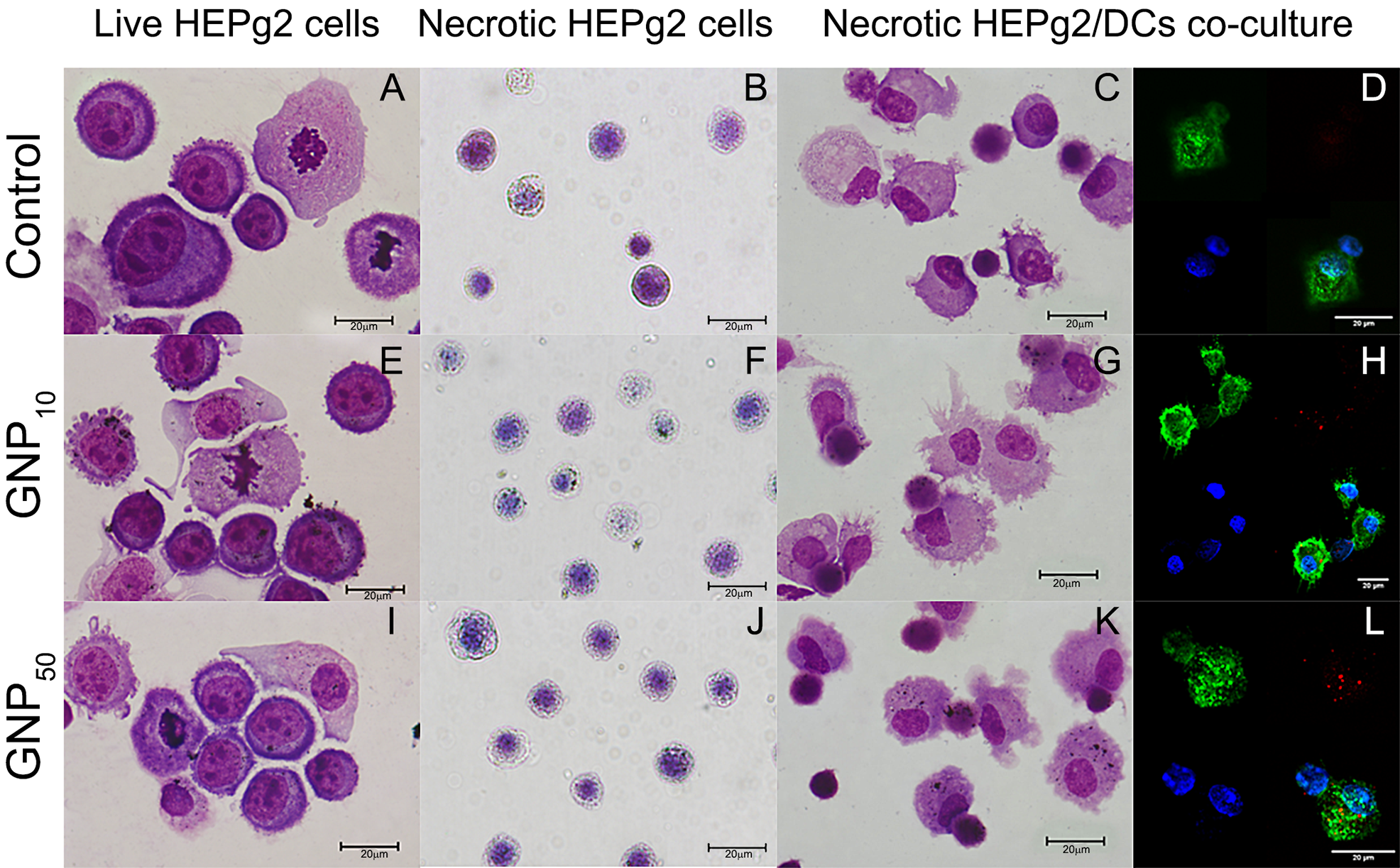

Supplement: Figure S4 — Co-cultures of GNP-loaded necrotic HEp-2 cells and DCs. HEp-2 cells were cultivated for 24 h with GNPs (10 µg/ml), harvested and analyzed after staining with MGG by light microscopy (A, E and I). The cells were then heat killed, as described in Methods, and analyzed by light microscopy after staining with Trypan blue solution (B, F and J). The necrotic tumor cells were co-cultivated with immature DCs for 48 h, followed by cell harvesting and preparation of cytospins. The samples were then stained with MGG (C, G and K), or with HLA-DR: Alexa-488 and PI and analyzed by confocal microscopy (D, H and L). GNPs in those experiments were detected by strong light scattering properties wtih 660/30 nm detector. (TIF) [file pone.0096584.s004.tif]
