# Supplementary material for: Size-Dependent Effects of Gold Nanoparticles Uptake on Maturation and Antitumor Functions of Human Dendritic Cells In Vitro
Source: PLoS One. 2014 May 6;9(5):e96584. doi: 10.1371/journal.pone.0096584 (PMC4011871; doi:10.1371/journal.pone.0096584)
Supplement: Table S1 — Cytokines production by LPS-treated DCs (IL-10, IL-12 and IL-23), and by CD4+T cells (IL-4, IFN-γ, IL-17) in subsequent co-culture. Summarized results are presented as median (range) of all experiments performed. (DOC) [file pone.0096584.s005.doc]

| **Table S1. Cytokines production by LPS-treated DCs (IL-10, IL-12 and IL-23), and by**  **CD4^+^T cells (IL-4, IFN-γ, IL-17) in subsequent co-culture.** | | | | | | |
| --- | --- | --- | --- | --- | --- | --- |
| **Median**  **(range)** | **IL-10** | **IL-12** | **IL-23** | **IL-4** | **IFN-γ** | **IL-17** |
| ***Control** | 68.9  (4.4-122.0) | 3.4  (<2.0-4.2) | 60.1  (<2-87.2) | 140.6  (20.6-169.9) | 7909.2  (3220.0-18900.1) | 328.8  (81.9-621.4) |
| ***GNP_10_** | 49.8  (4.6-106.5) | 10.7  (<2.0-14.5) | 40.4  (<2-99.6) | 110.2  (24.6-150.1) | 5989.0  (2991.4-15889.9) | 391.1  (65.5-832.2) |
| ***GNP_50_** | 55.5  (5.2-95.0) | 27.7  (<2-55.5) | 65.3  (<2-98.1) | 111.6  (25.0-158.4) | 9498.1  (4211.1-19844.5) | 411.4  (95.4-669.0) |
| **LPS** | 113.1  (9.8-169.3) | 4447.1  (128.4-8522.4) | 656.3  (46.1-1350) | 45.4  (10.4-105.9) | 29399.4  (8467.7-38299.0) | 555.1  (85.4-1159.4) |
| **LPS/GNP_10_** | 242.2  (11.9-434.3) | 1618.4  (4.5-3067) | 482.7  (9.0-702.0) | 120.1  (16.3-141.9) | 24399.2  (7546.1-32440.9) | 328.9  (64.3-723.3) |
| **LPS/GNP_50_** | 105.9  (<2-228.2) | 3992.2  (102.7-8163.6) | 920.7  (10.1-1640.0) | 49.4  (7.8-64.2) | 32838.2  (9122.1-38991.7) | 740.4  (141.9-975.0) |
| **HEp-2** | 38.1  (14.4-110.3) | 4.1  (<2-8.3) | 17.6  (12.9-32.2) | 190.9  (66.9-243.9) | 6144.5  (3780.0-19224.4) | 152.5  (54.7-435.4) |
| **HEp-2/GNP_10_** | 58.3  (17.4-124.3) | 4.5  (<2-9.1) | 47.8  (35.9-73.9) | 359.0  (121.2-445.5) | 5167.5  (2441.1-22134.4) | 425.3  (247.9-718.2) |
| **HEp-2/GNP_50_** | 45.4  (15.8-90.2) | 3.3  (<2-8.2) | 31.2  (20.7-59.3) | 215.2  (54-250.4) | 5898.8  (3909.4-17332.0) | 319.4  (99.8-549.4) |
| * Data pooled from 8 independent experiments, whereas other data was pooled from 4 independent experiments | | | | | | |
