# Supplementary material for: Size-Dependent Effects of Gold Nanoparticles Uptake on Maturation and Antitumor Functions of Human Dendritic Cells In Vitro
Source: PLoS One. 2014 May 6;9(5):e96584. doi: 10.1371/journal.pone.0096584 (PMC4011871; doi:10.1371/journal.pone.0096584)
Supplement: Table S2 — Interference of GNPs with the effect of LPS on phenotypic maturation of DCs. GNP10 and GNP50 (10 or 50 µg/ml) were incubated with LPS (100 ng/ml) in complete RPMI medium for 48 h, after which the supernatant was isolated by centrifugation, and the pellet was washed two times in complete medium. DCs were cultivated in the supernatant or in washed pellet preparation for 48 h, and the expression of indicated markers was measured by flow cytometry. Results are shown as mean ± SD of two independent experiments. *p<0.05 compared to control. (DOCX) [file pone.0096584.s006.docx]

| **Table S2. Interference of GNPs with the effect of LPS on phenotypic maturation of DCs** | | | | | |
| --- | --- | --- | --- | --- | --- |
|  | **LPS** | **LPS/GNP_10_** | | **LPS/GNP_50_** | |
|  | **100 ng/ml** | **10 μg/ml** | **50 μg/ml** | **10 μg/ml** | **50 μg/ml** |
| **Supernatant** |  |  |  |  |  |
| HLADR (MFI) | 66.5 ± 4.3 | 64.0 ± 3.4 | 65.6 ± 2.2 | 63.5 ± 2.1 | 59.5 ± 3.3 |
| CD86 (%) | 95.7 ± 2.4 | 93.1 ± 1.1 | 91.2 ± 2.1 | 91.5 ± 3.2 | 92.5 ± 2.2 |
| CD83 (%) | 55.6 ± 4.5 | 52.3 ± 4.3 | 48.6 ± 3.3 | 51.0 ± 2.5 | 44.3 ± 3.0* |
| **Washed pellet** |  |  |  |  |  |
| HLADR (MFI) | 22.3 ± 3.0 | 28.7 ± 4.3 | 27.5 ± 3.8 | 21.5 ± 3.0 | 24.3 ± 4.2 |
| CD86 (%) | 41.5 ± 2.2 | 43.3 ± 1.7 | 40.5 ± 2.7 | 44.5 ± 1.8 | 38.5 ± 3.0 |
| CD83 (%) | 22.7 ± 4.0 | 28.7 ± 3.6 | 25.2 ± 3.1 | 26.7 ± 3.6 | 32.3 ± 3.9* |

GNP_10_ and GNP_50_ (10 or 50 μg/ml) were incubated with LPS (100 ng/ml) in complete RPMI medium for 48h, after which the supernatant was isolated by centrifugation, and the pellet was washed two times in complete medium. DCs were cultivated in the supernatant or in washed pellet preparation for 48h, and the expression of indicated markers was measured by flow cytometry. Results are shown as mean ± SD of two independent experiments. *p<0.05 compared to control (Friedman’s two way ANOVA).
